# Molybdenum disulfide (MoS_2_) along with graphene nanoplatelets (GNPs) utilized to enhance the capacitance of conducting polymers (PANI and PPy)†

**DOI:** 10.1039/d3ra04153k

**Published:** 2023-10-02

**Authors:** Saima Nawaz, Yaqoob Khan, Sadia Khalid, Mohammad Azad Malik, Muhammad Siddiq

**Affiliations:** a Department of Chemistry, Quaid-i-Azam University Islamabad 45320 Pakistan m_sidiq12@yahoo.com +92 5190642147; b Nanoscience and Technology Department, National Centre for Physics, QAU Campus Shahdra Valley Road Islamabad 45320 Pakistan; c Department of Chemistry, University of Zululand Private Bag X1001 KwaDlangezwa 3880 South Africa malikmohammad187@gmail.com +44 7403781143

## Abstract

Hybrid composites of molybdenum disulfide (MoS_2_), graphene nanoplatelets (GNPs) and polyaniline (PANI)/polypyrrole (PPy) have been synthesized as cost-effective electrode materials for supercapacitors. We have produced MoS_2_ from molybdenum dithiocarbamate by a melt method in an inert environment and then used a liquid exfoliation method to form its composite with graphene nanoplatelets (GNPs) and polymers (PANI and PPy). The MoS_2_ melt/GNP ratio in the resultant composites was 1 : 3 and the polymer was 10% by wt. of the original composite. XRD (X-ray diffraction analysis) confirmed the formation of MoS_2_ and SEM (scanning electron microscopy) revealed the morphology of the synthesized materials. The electrochemical charge storage performance of the synthesized composite materials was assessed by cyclic voltammetry (CV), electrochemical impedance spectroscopy (EIS) and galvanostatic charge/discharge (GCCD) measurements. Resultant composites showed enhanced electrochemical performances (specific capacitance = 236.23 F g^−1^, energy density = 64.31 W h kg^−1^ and power density = 3858.42 W kg^−1^ for MoS_2_ melt 5 mPP at a current density of 0.57 A g^−1^ and had 91.87% capacitance retention after 10 000 charge–discharge cycles) as compared to the produced MoS_2_; thus, they can be utilized as electrode materials for supercapacitors.

## Abstract 2

Molybdenum disulfide (MoS_2_), along with graphene nanoplatelets (GNPs), is utilized to enhance the capacitance of conducting polymers (PANI and PPy) to produce cost-effective composite electrode materials for supercapacitors.

## Graphical abstract

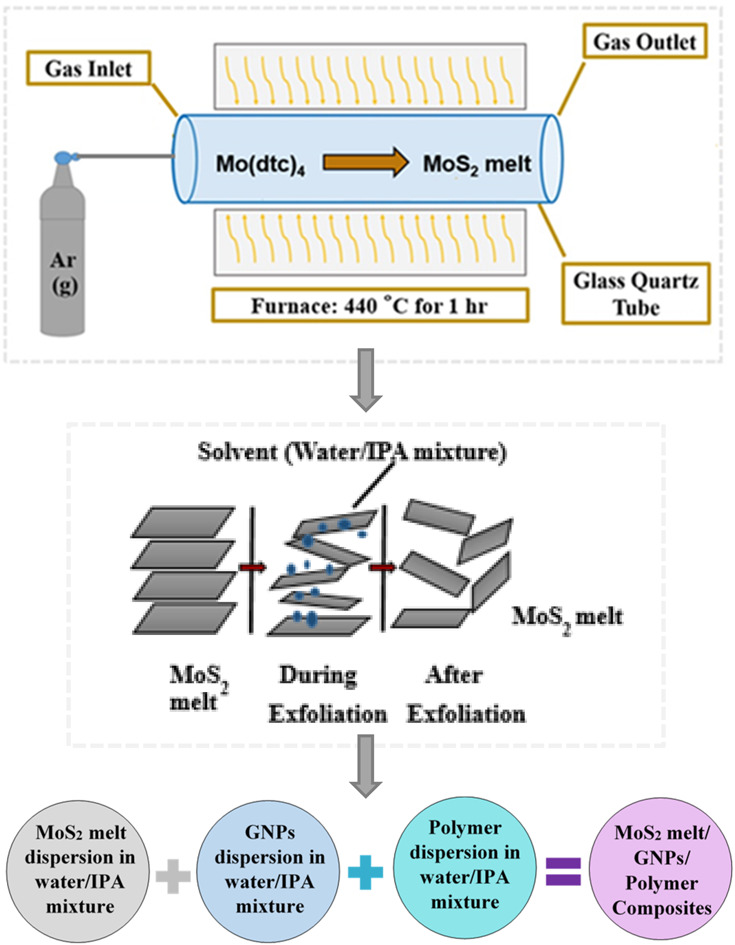

## Introduction

Supercapacitors are the next generation of energy storage devices, which bridge the gap between capacitors and batteries. Due to the quick charging and discharging rates of energy storage while having enhanced power density, excellent long cycle life and intermediate specific energy, supercapacitors have huge potential to replace batteries in the energy sector. They are a better fit for work environments where the equipment need urgent power for a brief period, such as in electric automobiles (*e.g.*, in emergency doors on the Air-bus A380). Supercapacitors are categorized into two types based on the mechanism of charge storage: pseudocapacitors and electric double-layer capacitors (EDLCs). Materials having energy storage as a consequence of quick and reversible redox phenomena fall under the category of pseudocapacitors, *e.g.*, conducting polymers and oxides of transition metals. The energy storage mechanism of EDLCs is mainly an ion absorption phenomenon occurring at the electrical double layer present at the electrode/electrolyte interface. Carbon-based materials possessing higher surface areas are the most used ingredients in the supercapacitor domain and are included in EDLCs, which display lower specific capacitance in comparison to pseudocapacitors.^1–10^ Faradaic pseudocapacitors are typically categorized into two classes, *i.e.*, redox pseudocapacitance (redox reactions occur at the surface/near-surface) and intercalation pseudocapacitance (counter-ions of electrolyte intercalate inside layers existing in the active material of the electrode along with faradaic charge-transfer without any phase transitions).^11,12^

To accomplish better electrochemical performance, various chemical and structural changes have been made to traditional materials that were previously used in the supercapacitor industry or the hybridization of these materials is done, resulting in hybrid composite systems with the synergistic effects of each constituent material. These resultant nanocomposites/hybrids integrate the beneficial characteristics and compensate for the disadvantageous attributes linked with each component. Carbon and transition metal (oxides, hydroxides and dichalcogenides)-based materials are among the most studied materials for the active ingredients of electrodes for the supercapacitor domain.^13^

Carbon and its allotropes possess superior properties of larger surface area-to-weight ratios, higher electrical conductivities, better electrochemical stability and long cycle lives and, therefore, are widely used materials for electrodes of EDLC supercapacitors. From activated carbon to carbon nanotubes (CNTs), mesoporous carbon and hierarchical templated carbons, many evolutionary studies are being conducted to achieve better electrochemical storage performance.^14^ Graphene, being expensive, and reduced graphene oxide, being of relatively high cost and having significant defects in structure even after reduction, are not so promising as reinforcements of composites.^15^ Graphene nanoplatelets (GNPs) have emerged as the most promising, attractive and ideal reinforcing material because of their superior properties such as low cost, high structural integrity, high aspect ratio, extraordinary mechanical properties, and good electrical and thermal conductivity.^16^ The era of GNPs/polymer hybrid nanocomposites has just begun and appears to be a very small piece of a huge cake due to the cost-effective GNPs that have the benefits of reduced graphene oxide, carbon nanotubes and silicate layers.^15^ GNPs can transform plastic into an electrical capacitor, making it the ideal material for electronic devices.^17^

In contrast, pseudo-capacitive materials for electrodes of supercapacitors including some transition metal dichalcogenides (TMDs) and conducting polymers (CPs) store more electrochemical energy due to fast reversible redox phenomena (surface-controlled).^18^ TMDs typically exhibit large theoretical capacitance and higher specific surface area, which have been extensively researched in the field of high-performance supercapacitors.^19–22^ Among several TMDs, molybdenum disulfide (MoS_2_) is regarded as an exceptional electrode material. MoS_2_ has a layered structure akin to graphene and covalent bonding of S–Mo–S *via* van der Waals interactions inside its system, Pt-like electronic features and relatively high theoretical specific capacitance.^23–25^ High-performance charge storage and conversion devices require higher electron transfer efficiency and better electrolyte infiltration, both of which are made possible by the layered structure.^26–28^ However, weak conductivity along with a lack of sufficient electrochemically active sites severely limits its practical applicability. It has been proven by previous reports that doping of heteroatoms in the structure and phase engineering of MoS_2_ are efficient approaches to getting around its intrinsic constraints and ultimately improving its electrocatalytic and electrochemical charge storage performance.^25^

Among the several CPs, polyaniline (PANI) and polypyrrole (PPy) are notably appealing options because of their simple synthesis, environmental stability and controllable higher electrical conductivity (due to the presence of delocalized p-electrons along the backbone in its conjugated structure).^29,30^ Being a pseudocapacitive electrode material with a high theoretical specific capacitance carries several benefits. They also have short ionic transport path lengths that enable quicker ionic diffusion inside the network of the polymer due to which energy is ultimately supplied very quickly.^31^ The interaction of a large surface area of CPs with the electrolyte material enables relatively faster rates of charging and discharging. However, there is a disadvantage of volume change (swelling and shrinking) associated with the phenomena of doping-dedoping, which results in much reduced mechanical attributes and low cycling stability when CPs are utilized as the electrode material on their own (*i.e.* without any doping/hybridization) for supercapacitors.^32,33^ Sonication and irradiation techniques during manufacturing or hybridization with different types of fillers are among the methods employed to address this problem. These methods ultimately increase the volume of CPs, provide room for their swelling and improve porosity.^31^ Previous reports confirmed that compositing CPs with other materials (*e.g.* carbon-based materials, inorganic sulfides, oxides and hydroxides and various other metal compounds) enhances the capacitance and improves the cyclic stability of resultant composites.^6,31,34–41^

The novelty of this research is mainly the synthesis of hybrid composites of MoS_2_, GNPs and PANI/PPy as the cost-effective electrode materials for supercapacitors. The objective is to assess the effects of all constituent materials of the composites on the overall charge storage performance of the electrode. In this work, we produced MoS_2_ from molybdenum dithiocarbamate by a melt method in an inert environment and then used a liquid exfoliation method to form its composite with graphene nanoplatelets (GNPs) and polymers (polyaniline and polypyrrole). The resultant composites showed enhanced electrochemical performances as compared to the produced MoS_2_ so they can be utilized as electrode materials for supercapacitors.

## Experimental

### Materials

Analytical-grade chemical reagents were obtained from Sigma-Aldrich and were employed directly without any further modification. Graphene nanoplatelets, *i.e*., GNPs (M5 *i.e.* Grade M with average particle diameters of 5 microns) were purchased from XG Sciences.

#### Synthesis of tetrakis(diethyldithiocarbamato)molybdenum(iv) (Mo(dtc)_4_)

The synthetic technique of Decoster *et al.* was utilized to prepare Mo(dtc)_4_.^42^ In short, molybdenum hexacarbonyl and tetraethylthiuram disulfide were heated to reflux in acetone and the resultant mixture was kept at this temperature for 2 hours. To attain the solid crystals, this mixture was allowed to slowly cool, uninterrupted for an hour, to ambient temperature. The obtained crystalline material product was separated from by-products by vacuum filtration and the subsequent washing with pentane.

#### Synthesis of MoS_2_ from Mo(dtc)_4_

**Fig. 1:**
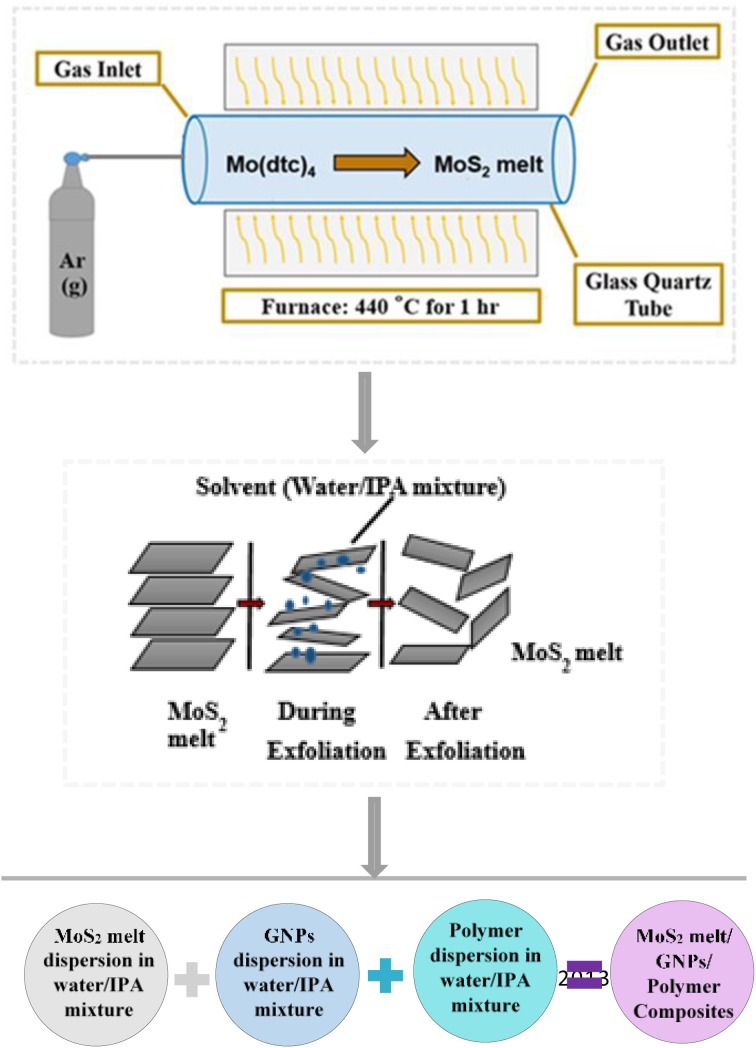
Schematic diagram showing the synthesis of MoS_2_ melt/GNPs/polymer composites.

Mo(dtc)_4_ powder was placed in a ceramic boat and decomposed by heating at 440 °C in the centre of a Carbolite MTF furnace for one hour under an inert environment of argon to synthesize the MoS_2_ melt (Fig. 1). The final product, a black powder, was attained after the system was allowed to cool to ambient temperature.^43^

#### Synthesis of polyaniline

In a typical synthesis, 0.02 moles of aniline dissolved in a 0.02 M HCl solution were added to the aqueous solution of cetyltrimethylammonium bromide (CTAB) (0.00125 mol) with constant stirring at 4–5 °C. Later, a 0.04 M ice-cold aqueous solution of ammonium persulfate (APS, *i.e.*, oxidizing agent) was slowly added to the reaction mixture to attain a homogeneous mass. The resultant mixture was kept under static conditions for 2 days at 0 °C to age. The resultant mesoporous polyaniline polymer (mP) was thoroughly filtered/washed using deionized water and ethanol solvent and then ultimately dried at ambient temperature under vacuum conditions.^44^

#### Synthesis of polypyrrole

Here, 0.0045 mol of surfactant (CTAB) was dissolved in 20 mL of distilled water. Pyrrole monomer (0.05 M = 3.47 mL) was then added and mixed by constant stirring for 15 min to make a homogeneous solution. The oxidant, *i.e.*, 0.1 M aqueous solution of FeCl_3_ (20 mL), was added slowly to the monomer-surfactant solution and kept at a fixed temperature of 0 °C with stirring; the polymerization time was about 20 min and the solution eventually transitioned from yellow to dark brown. The reaction product was filtered and then washed with methanol until a clear filtrate was achieved the obtained sample was dried under a vacuum for more than 12 hours at room temperature. The resulting mesoporous polypyrrole (mPP) was a black powder.^45^

#### Synthesis of MoS_2_ melt/GNP/polymer (mP, and mPP) composites

All dispersions were prepared by using the technique of liquid phase exfoliation as reported earlier.^46–48^ The MoS_2_ melt (10 mg/10 mL) was dispersed in an IPA (isopropanol)/water (1 : 1) mixture and then sonicated for 1 h in an Elmasonic P70H sonicator (frequency = 37 kHz, amplitude = 40%) to ensure homogeneous mixing while cooling to maintain the temperature at 20 °C. Subsequently, to remove any unexfoliated material from the final dispersion, centrifugation was conducted for 30 min at 6000 rpm (3139 g). GNPs (M5) and polymer (mP or mPP) were dispersed separately in an IPA (isopropanol)/water (1 : 1) mixture in the same manner as used for the MoS_2_ melt.

The three resultant dispersions (MoS_2_ melt dispersion, M5 dispersion and polymer dispersion) were combined and sonicated for another hour and the solvent was evaporated to obtain the dried composite. The composite with mP was designated as MoS_2_ melt 5 mP and that with mPP was named MoS_2_ melt 5 mPP. The MoS_2_ melt/GNPs ratio in the resultant composite was 1 : 3 and the polymer was 10% by wt. of the original composite (Fig. 1).

### Characterizations

XRD diffraction patterns of the synthesized samples were obtained *via* a Bruker D8 Advance diffractometer. SEM was conducted using a field emission microscope of Hitachi Su-70 Schottky. Using the KBr pellet method at room temperature in the 400–4000 cm^−1^ range, FT-IR spectra were obtained on a Bio-Rad Excalibur FT-IR Spectrometer. The microanalytical lab at the University of Manchester carried out elemental studies by employing a Thermo Scientific Flash 2000 organic elemental analyzer. Thermogravimetric analysis was conducted utilizing a Seiko SSC/S200 model in an environment of nitrogen while maintaining a heating rate of 10 °C min^−1^. Raman spectra were obtained on a Renshaw 1000 system, utilizing a 514.5 nm solid-state laser (operating at 10% power). A 50× objective lens was utilised to focus the laser beam onto the synthesized samples. An air-cooled charge-coupled device (CCD) detector was used to detect the scattered signal.

### Electrochemical measurements

A three-electrode system along with an electrolyte of 1 M H_2_SO_4_ aqueous solution was utilized to study the electrochemical characteristics of the synthesized electrode materials. A saturated calomel electrode (SCE) was used as the reference electrode, while a graphite rod was used as the counter electrode. Polytetrafluoroethylene (PTFE), which serves as the binder, was combined with active materials to prepare the working electrodes. Cyclic voltammetry (CV), electrochemical impedance spectroscopy (EIS), and galvanostatic charge/discharge (GCCD) data were gathered by employing the Gamry REF3000 electrochemical workstation. At various scan rates, scans of CV were obtained within a potential window of −0.4–1 V. Moreover, at the scan rate of 20 mV s^−1^, the cyclic stability of each electrode material was tested for 50 cycles. By integrating the CV curve to obtain the average area under the curve for one complete CV cycle, the specific capacitance (*C*_sp_) can be computed by using the relation^49^ given in eqn (1):1
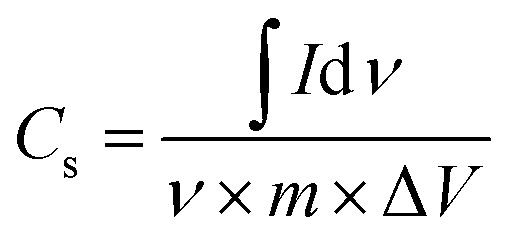
where ∫*I*d*ν* = the total integrated area of the curve, *ν* = scan rate, Δ*V* = potential window, *m* = mass of the active material of the electrode.

By employing the formula^50^ given in eqn (2), one can also estimate the specific capacitance of a three-electrode system using the obtained slope of the discharge curve from GCCD measurements.2
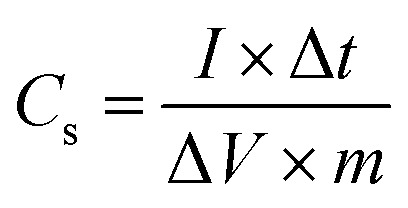
where Δ*V*/Δ*t* = the slope of the discharge curve, *I* = discharge current, *m* = the mass of the active material of the electrode.

## Results and discussion

### X-ray diffraction (XRD) analysis

**Fig. 2:**
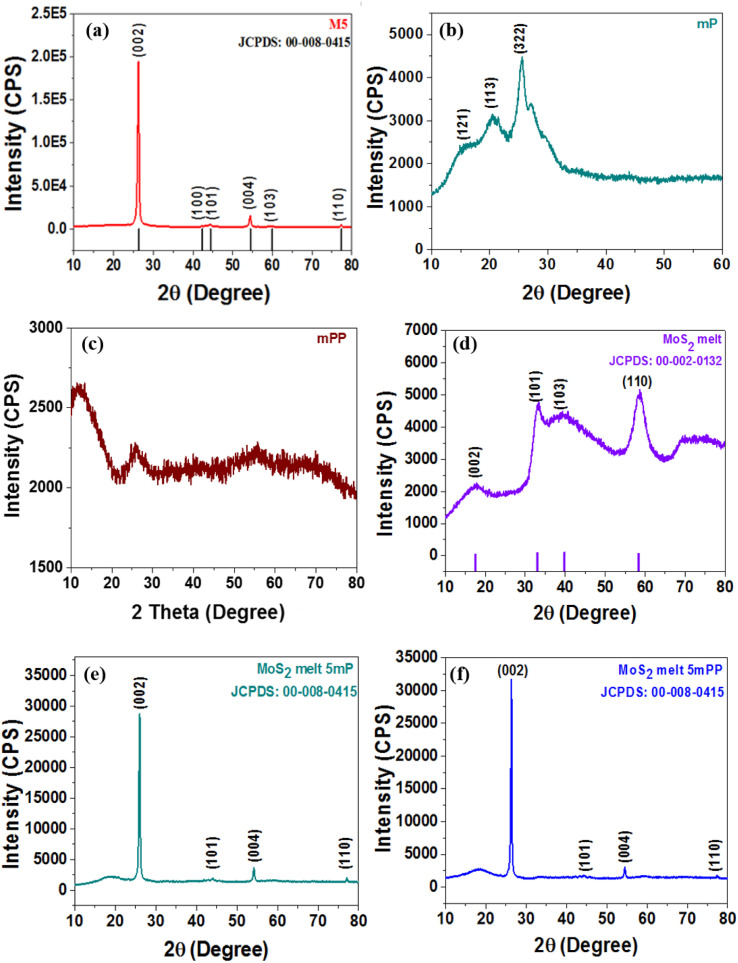
X-ray diffraction (XRD) patterns of (a) M5, (b) mP, (c) mPP, (d) MoS_2_ melt, (e) MoS_2_ melt 5 mP and (f) MoS_2_ melt 5 mPP.

To examine the crystallographic structure of the synthesized electrode materials, X-ray powder diffraction (XRPD) analysis was executed and XRD patterns are shown in Fig. 2. The diffraction pattern of the MoS_2_ melt (Fig. 2d) corresponds to standard 2H-MoS_2_ (JCPDS no. 00-002-0132). The peaks related to the (101) and (110) diffraction planes of the MoS_2_ melt were rather intense but those associated with the (002) and (103) planes were significantly broadened (Fig. 2d). The pattern indicates that MoS_2_ crystallites might grow laterally in the phenomenon of thermolysis since the planes of (101) and (110) seem to be the preferred growth orientation. There is reduced bulk character in the direction of [00*l*], as evidenced by the weak reflection of the (002) diffraction plane. This again demonstrated that it was possible to synthesize MoS_2_ sheets with nanoscale thickness *via* this chemical reaction pathway. The mP (polyaniline) in Fig. 2b, displays the diffraction peaks at phase angles of 14.4°, 19.6°, and 25.4°, which are associated with the reflections of the (121), (113) and (322) planes, respectively, indicating that mP is in the emeraldine salt form.^51,52^Fig. 2c (mPP) shows the characteristic amorphous peaks of typical polypyrrole prepared by using FeCl_3_ as the oxidant at 12.0°, and 26.1°, which shows that the synthesized mPP (polypyrrole) is amorphous. This was also reported in earlier literature.^53,54^ Such broad peaks are usually due to the short-range arrangement of polymer chains.

The (002), (100), (101), (004), (103) and (110) crystal planes of M5-GNPs respectively produced peaks at 2*θ* = 26.3°, 43.3°, 44.3°, 54°, 59.7° and 77.3° (JCPDS no. 00-008-0415) visible in the XRD pattern of M5 (Fig. 2a). The XRD patterns of the composites, MoS_2_ melt 5 mP (Fig. 2e) and MoS_2_ melt 5 mPP (Fig. 2f), mainly showed the peaks of M5-GNPs (which is also the major component of the composite electrode material) and the characteristic peaks for PANI/PPy and MoS_2_ melt seemed to be suppressed (disappeared) in the presence of the high-intensity peaks of M5. M5 is highly crystalline and there is a greater amount in composites as compared to MoS_2_ and polymers.

### Scanning electron microscopy (SEM)

**Fig. 3:**
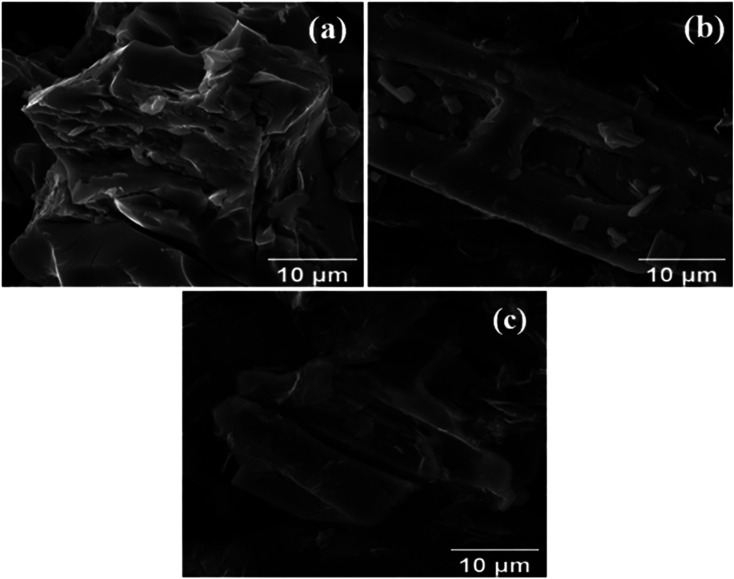
Scanning electron microscopy (SEM) images of the (a) MoS_2_ melt, (b) MoS_2_ melt 5 mP and (c) MoS_2_ melt 5 mPP.

Fig. 3a displays the SEM image of the MoS_2_ melt, showing large crystallites embedded in the surface of an agglomerated mass formed by the accumulation of MoS_2_. SEM images of MoS_2_ melt 5 mP (Fig. 3b) and MoS_2_ melt 5 mPP (Fig. 3c) show uneven distribution of crystallite along GNPs. The overall appearances of both composites, MoS_2_ melt 5 mP (Fig. 3b) and MoS_2_ melt 5 mPP (Fig. 3c), are different, which might be due to the effects of polymers, *i.e.*, mP and mPP, respectively, although no significant appearance of polymers can be seen in these images.

### Electrochemical studies

#### Cyclic voltammetry (CV)

##### CV at various scan rates

**Fig. 4:**
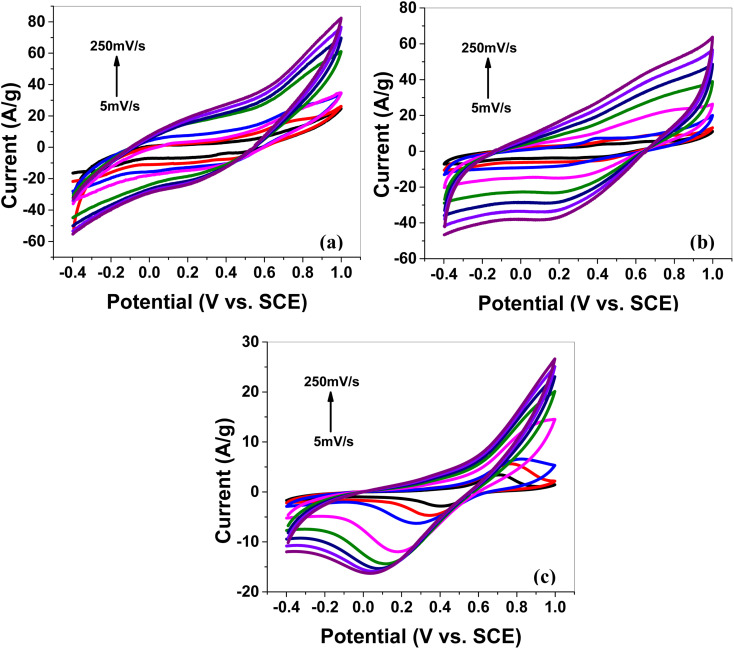
Cyclic voltammetry (CV) of (a) the MoS_2_ melt 5 mPP, (b) MoS_2_ melt 5 mP and (c) MoS_2_ melt at different scan rates on GCE in 1 M H_2_SO_4_*vs.* SCE.

Cyclic voltammetry (CV) was performed in a 1 M aqueous solution of H_2_SO_4_ as an electrolyte to study the electrochemical storage performance of the synthesized electrode samples. The CV scans (Fig. 4a–f,) for all synthesized electrode materials (MoS_2_ melt 5 mPP, MoS_2_ melt 5 mP, MoS_2_ melt, M5, mPP and mP) were executed in the potential window of −0.4 to 1 V (*versus* SCE). The CV for each electrode material was processed at various scan rates in the range of 5 to 250 mV s^−1^ (*i.e.*, 5, 10, 20, 50, 100, 150, 200 and 250 mV s^−1^).

The cyclic voltammogram of MoS_2_ melt 5 mPP (Fig. 4a) showed the characteristic square-shaped curve, which is indicative of EDLC at lower scan rates with no evident pseudocapacitance peaks. However, at higher scan rates, the curves diverged from the square shape and showed a combination of square and redox peaks, which implies the changed mechanism of charge storage to fast redox reactions due to pseudocapacitance.^55–59^

The specific capacitance decreases rapidly on increasing the scan rate, which also reflects the change in the storage mechanism of charge. The relatively slow phenomenon of ion adsorption contributes to the overall capacitive behavior of the electrode dominating at low scan rates but as the speed of the charging/discharging processes increases, a faster phenomenon also occurs, resulting in a lower double-layer capacitance value. This has been observed many times in earlier research on supercapacitors associated with transition metal dichalcogenides (TMDCs).^60–63^

Eqn (3) can be used to describe a non-faradaic process based on the phenomena of H^+^ ion adsorption and desorption on the surface and intra-sheets of MoS_2_, forming a double layer at the interface of the electrolyte/electrode material.

3 (MoS_2_)_surface_ + *x*H^+^ + *x*e^−^ ↔ H_*x*_ (MoS_2_)_surface_

The cyclic votammetry peaks of the MoS_2_-based electrode could be assigned to the redox phenomenon (given in eqn (4)) of insertion/extraction of H^+^ into/from the layered structure of the MoS_2_ material (*i.e.* Mo-IV ↔ Mo-V ↔ Mo-VI).^55,64–66^

4 MoS_2_ + *x*e^−^ + *x*H^+^ ↔ H_*x*_ (MoS_2_)

The MoS_2_ melt 5 mPP composite (Fig. 4a) showed the same electrochemical response as the MoS_2_ melt 5 mP (Fig. 4b) and MoS_2_ melt (Fig. 4c), but the peak current of the MoS_2_ melt 5 mPP increased significantly, indicating a higher capacitance of the electrode. The enhanced electrochemical utilization of MoS_2_ melt 5 mPP is due to the availability of more electroactive sites as a consequence of the favourable configuration of the hybrid nanocomposite for electrochemical storage phenomena.

By comparing the shapes of the cyclic voltammograms of MoS_2_ melt 5 mPP, MoS_2_ melt 5 mP, and MoS_2_ melt (Fig. 4a–c), it was observed that the contribution of the MoS_2_ melt to the total capacitance of the composite electrodes of MoS_2_ melt 5 mPP and MoS_2_ melt 5 mP was of the double-layer type, which is also evident from the CV curves of the MoS_2_ melt (Fig. 4c). The cyclic voltammogram of M5 (Fig. S4a†) shows the characteristic square-shaped curve, which is indicative of EDLC behavior while CV curves of mPP (Fig. S4b†) and mP (Fig. S4c†) show typical pseudocapacitance behavior. Therefore, M5 contributed diffusion-controlled capacitance and mPP/mP contributed pseudocapacitance to the total capacitance achieved in the case of the hybrid composite of the MoS_2_ melt 5 mPP/MoS_2_ melt 5 mP. The GNPs (M5) are higher in composites so their contribution is greater, while that of the polymer (mPP/mP) is less so its pseudocapacitive contribution is less.

The electrochemical behavior of MoS_2_ melt 5 mPP (Fig. 4a) is better than MoS_2_ melt 5 mP (Fig. 4b), which might be because in the hybrid composite form, the mPP along with GNPs and MoS_2_ melt provide a more favourable configurational structure for the flow of charge inside the material as compared to the hybrid composite of the mP along with GNPs and MoS_2_ melt.

CV curves for synthesized samples (Fig. 4a–c and S4a–c†) are rectangular and are mirror images, indicating high electrochemical reversibility. The specific capacitances (*C*_sp_) calculated using eqn (1) for the composite electrodes of MoS_2_ melt 5 mPP, MoS_2_ melt 5 mP, and MoS_2_ melt were 145.39, 91.23 and 33.06 F g^−1^, respectively, at the scan rate of 5 mV s^−1^ and 1.4 V potential. The *C*_sp_ of M5, mPP and mP are 75.48, 55.62 and 28.52 F g^−1^ respectively (potential = 1.4 V, scan rate = 5 mV s^−1^).

#### Cyclic stability for 50 cycles of CV

**Fig. 5:**
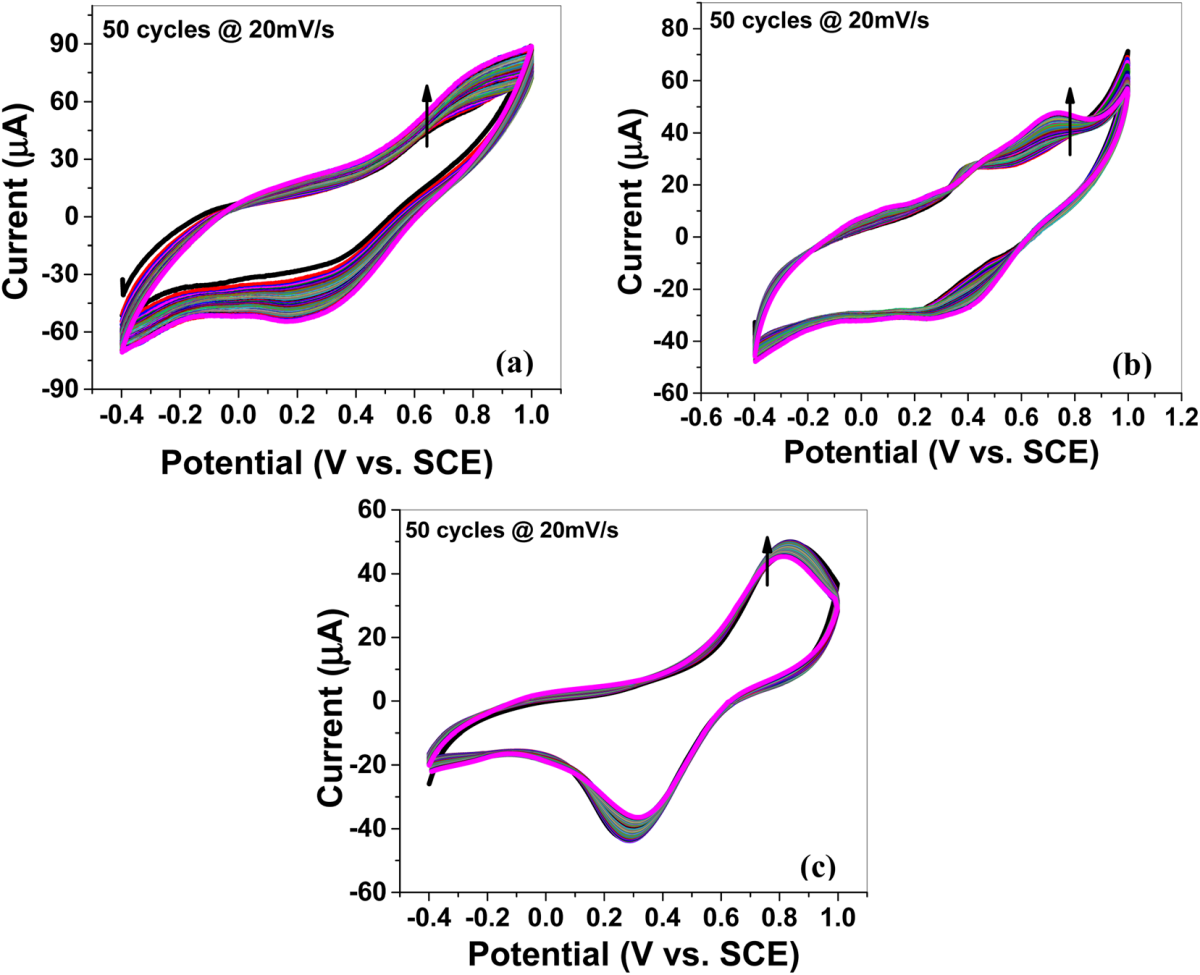
Cyclic voltammetry (CV): cyclic stability of the (a) MoS_2_ melt 5 mPP, (b) MoS_2_ melt 5 mP and (c) MoS_2_ melt at the scan rate of 20 mV s^−1^ for 50 cycles on GCE in 1 M H_2_SO_4_ electrolyte *vs.* SCE.

Fig. 5a–c and S5a–c† exhibit the cyclic voltammograms recorded in 1 M H_2_SO_4_ aqueous electrolyte solution for 50 cycles at 20 mV s^−1^ scan rate. The observed CV scans for 50 cycles were quite stable, and none of the electrode materials exhibited a discernible reduction in current, indicating that these materials have high cyclic stability.

#### Relative contribution to the total capacitance from capacitive or diffusion-controlled behavior

**Fig. 6:**
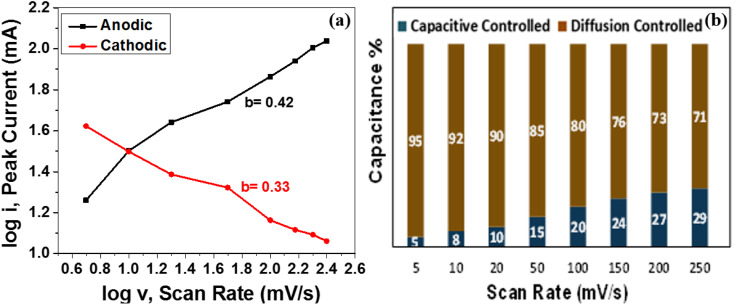
(a) Plot of log(*i*) *versus* log(*v*) for the anodic and cathodic current peaks of MoS_2_ melt 5 mPP (b) pseudocapacitive contribution (%) of MoS_2_ melt 5 mPP at different scan rates.

By utilising the power law^10,21,67–72^ (eqn (5)), it is possible to determine the relative contributions to the total capacitance from the surface or bulk mechanism. In this regard, the analysis of CV data at different scan rates (5–250 mV s^−1^) is displayed in Fig. 6a.

5 *i* = *av*^*b*^

where *i* = redox peak current, *v* = various scan rates.

According to the power law, the *b*-value attained from the slope of the plot of log(*i*) and log(*v*) may be used for the estimation of the governing energy storage mechanism of the active electrode material for supercapacitors. In the case of an electrode material being an ideal capacitor type, pseudocapacitive behaviour predominates and the value of *b* is near to 1. Whereas, for battery-type electrode materials, the *b*-value is close to 0.5 and has mainly diffusion-controlled behaviour that dominates as the charge storage mechanism. Fig. 6a depicts the plot of log(*i*) *versus* log(*v*) displaying cathodic and anodic peak currents for the MoS_2_ melt 5 mPP as shown in Fig. 4a. The *b*-value from the cathodic peak was 0.33, and that of the anodic peak was 0.42. This indicates that the resultant capacitance of the electrode material is primarily because of diffusion-controlled phenomenon and the MoS_2_ melt 5 mPP composite electrode displays mainly EDLC behaviour as the GNPs and MoS_2_ melt are the main constituents of hybrid composites so their storage mechanism is also dominant overall in the composite.^73–75^ By employing the Dunn method of differentiation,^76^ it was estimated that 5%, 8%, 10%, 15%, 20%, 24%, 27% and 29% of the total capacitance obtained for MoS_2_ melt 5 mPP is from the pseudocapacitive contribution and 95%, 92%, 90%, 85%, 80%, 76%, 73% and 71% is diffusion controlled behavior at scan rates of 5, 10, 20, 50, 100, 150, 200 and 250 mV s^−1^, respectively (Fig. 6b).

#### EIS (electrochemical impedance spectroscopy)

To evaluate the resistance of the electrochemical phenomena, EIS was performed (frequency = 10^6^–10^−1^ Hz, open circuit potential amplitude = 0.34, AC voltage = 10 mV rms and stabilization time = 10 s).

#### EIS Nyquist plots

**Fig. 7:**
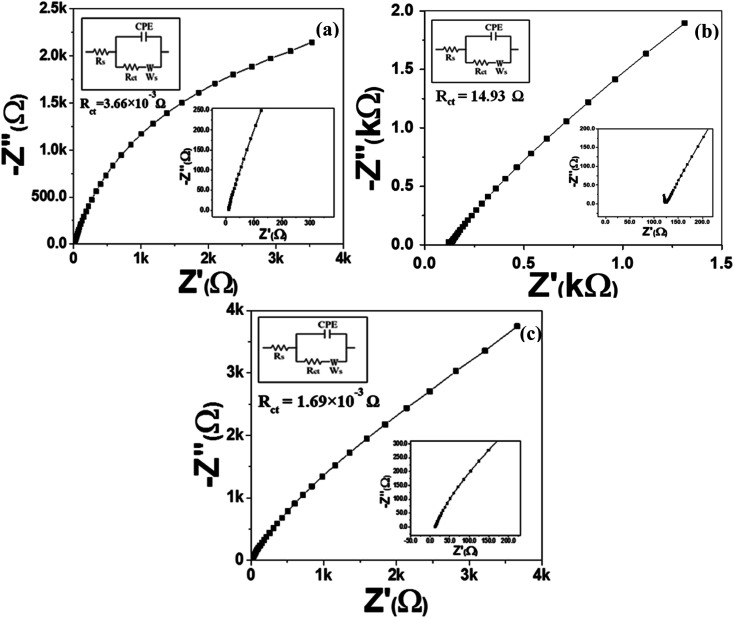
EIS Nyquist plots of (a) MoS_2_ melt 5 mPP, (b) MoS_2_ melt 5 mP and (c) MoS_2_ melt *vs.* OC in 1 M H_2_SO_4_ electrolyte, with equivalent circuit diagrams.

Fig. 7a–c and S6a–c† depict the Nyquist plots of all synthesized samples. Fig. 7a shows the frequency response of the MoS_2_ melt 5 mPP/1 M H_2_SO_4_ aqueous electrolyte system in the plot of real impedance (*Z*′) against imaginary impedance (*Z*′′). The equivalent circuit model (displayed in the inset of Fig. 7a–c and S6a–c†) is utilized to fit the Nyquist plot for each electrode material, where CPE, *W*, *R*_s_ and *R*_ct_ are the constant phase element, Warburg impedance, solution ohmic resistance and charge transfer resistance, respectively. The absence of a semicircle in the high-frequency region of Fig. 7a depicts a low value of *R*_ct_. The value of *R*_ct_ existing between the MoS_2_ melt 5 mPP composite electrode material and 1 M H_2_SO_4_ aqueous electrolyte is 3.66 × 10^−3^ Ω (attained by fitting equivalent circuit model) is significantly less, which might be because of the highly cross-linked conductive network of the mPP and MoS_2_ melt with GNPs. It may relate to the enhanced pseudocapacitance of mPP and shows that the surface properties are in favour of fast and facile charge transfer within the electrode material of the MoS_2_ melt 5 mPP composite.^77^

The higher impedance witnessed for the electrode material of MoS_2_ melt 5 mP in Fig. 7b indicated a high *R*_ct_ value (14.93 Ω), which may be because of a high hindrance to counter-ion flow inside the hybrid composite of MoS_2_/GNPs with mP as compared to that of mPP. The *R*_ct_ value for the MoS_2_ melt in Fig. 7c is 1.69 × 10^−3^ Ω.

The steeper curve due to the decreased Warburg impedance in Fig. 7a (MoS_2_ melt 5 mPP) at the low-frequency region illustrates the accelerated rate of adsorption and diffusion phenomena for the counter-ions of electrolytes in/on the electrode material.

The reason for this observation is that the hybrid network of the mPP and MoS_2_ melt with GNPs has more electroactive sites and provides more spaces with equal and smaller diffusion path lengths used for the transportation of counter-ions of 1 M H_2_SO_4_ aqueous electrolyte, subsequently resulting in enhanced electrochemical charge storage performance.

The Nyquist plot (Fig. 7b) for the MoS_2_ melt 5 mP showed a larger Warburg impedance as compared to the MoS_2_ melt 5 mPP (Fig. 7a), which might be due to greater variations in path lengths for ion diffusion in the hybrid composite of MoS_2_/GNPs with mP than that of mPP.

The value of the slope for each synthesized electrode (Fig. 7a–c and S6a–c†) declined as the AC frequency (potential) increased, showing an increased Warburg resistance that, in turn, cuts down the performance of the electrode for effective charge storage.

All electrodes of MoS_2_ melt 5 mPP, MoS_2_ melt 5 mP, MoS_2_ melt, M5, mPP and mP follow the equivalent circuit model of CPE with diffusion as shown in the inset of Fig. 7a–c and S6a–c.†

#### EIS bode plots

**Fig. 8:**
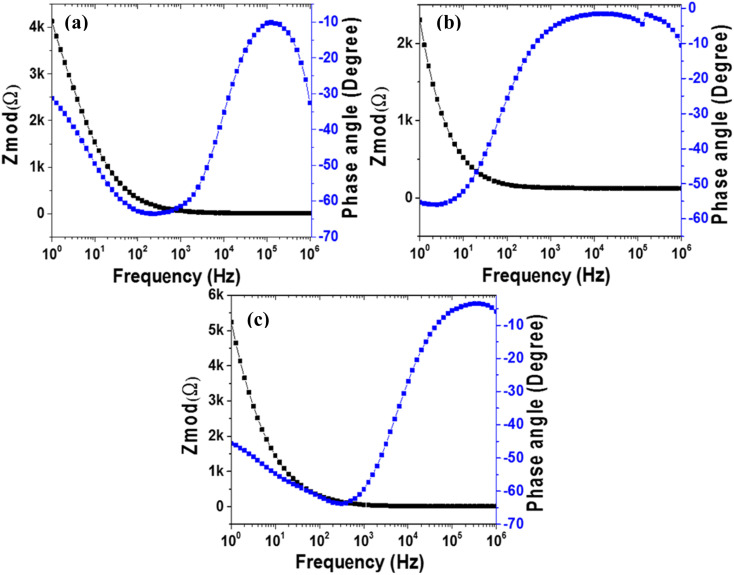
. EIS (electrochemical impedance spectroscopy): Bode plots of (a) MoS_2_ melt 5 mPP, (b) MoS_2_ melt 5 mP and (c) MoS_2_ melt *vs.* OC (open circuit) at 10 mV rms AC perturbation (in 1 M H_2_SO_4_ electrolyte).

Fig. 8a–c and S7a–c† show the Bode plots of MoS_2_ melt 5 mPP, MoS_2_ melt 5 mP, MoS_2_ melt, M5, mPP and mP respectively. In EIS Bode plots, the *x*-axis is the log of the frequency, and either impedance (|*Z*|) or the measured phase angle (*Φ*) is on the *y*-axis, and these two forms of plots are called the Bode magnitude plot” and “Bode phase angle plot”, respectively. Bode plots provide information regarding the corresponding change occurring in ion diffusion, resistance and capacitance with the effect of applied frequency.^78^ The phase angles in low-frequency domains from Bode phase angle plots provide specific information about the material of the electrode, which is expected to be −90° for an ideal capacitor, while for an ideal resistor, its value is zero (*Φ* = 0°). A phase angle near −45° in the low-frequency region is indicative of pseudocapacitance.^78–82^

The Bode plot of MoS_2_ melt 5 mPP in Fig. 8a shows the phase angle of −30.78 in the lower frequency region confirming the pseudocapacitive properties of the electrode.^78,82^ Bode plots of MoS_2_ melt 5 mP, MoS_2_ melt, M5, mPP and mP in Fig. 8a–c and S7a–c† show the phase angles of −55.29, −45.68, −57.90, −59.20 and −59.49, respectively in the lower frequency region.

Significant drops below the value of *Φ* = −90° indicate that the dielectric layer is less effective at holding electric charge and releases the flow of electrons. This would be because of a decrease in the total impedance as a consequence of current leakage occurring at defective sites present in the heterogeneous dielectric layer.^81^

The impedance of a capacitor only contains the *Z*′′ component and is inversely related to the frequency value, so with an increase in frequency, the value of impedance will decrease.^83,84^ A small value for the impedance (*i.e.* |*Z*|) in the low-frequency section can denote the decrease in resistance of the electrode material and an enhanced flow of electrons through the electrode,^85^ while a high value of |*Z*| can also indicate suitable capacitive and insulating properties.^78,86^ Bode magnitude plots of capacitive materials have slopes near −1,^87,88^ while a slope close to 0 is representative of resistive behavior that might occur in the domain of high frequencies for capacitive behavior.^89^

The impedance Bode plots (Fig. 8a–c and S7a–c†) of all samples showed high values for the slope in the low-frequency region, which is probably because of resistive behavior occurring at the interfaces; the lowest values of the slope in the high-frequency regions portray the purely capacitive nature of the electrode.^90,91^

#### Galvanostatic charge/discharge (GCCD) measurements

**Fig. 9:**
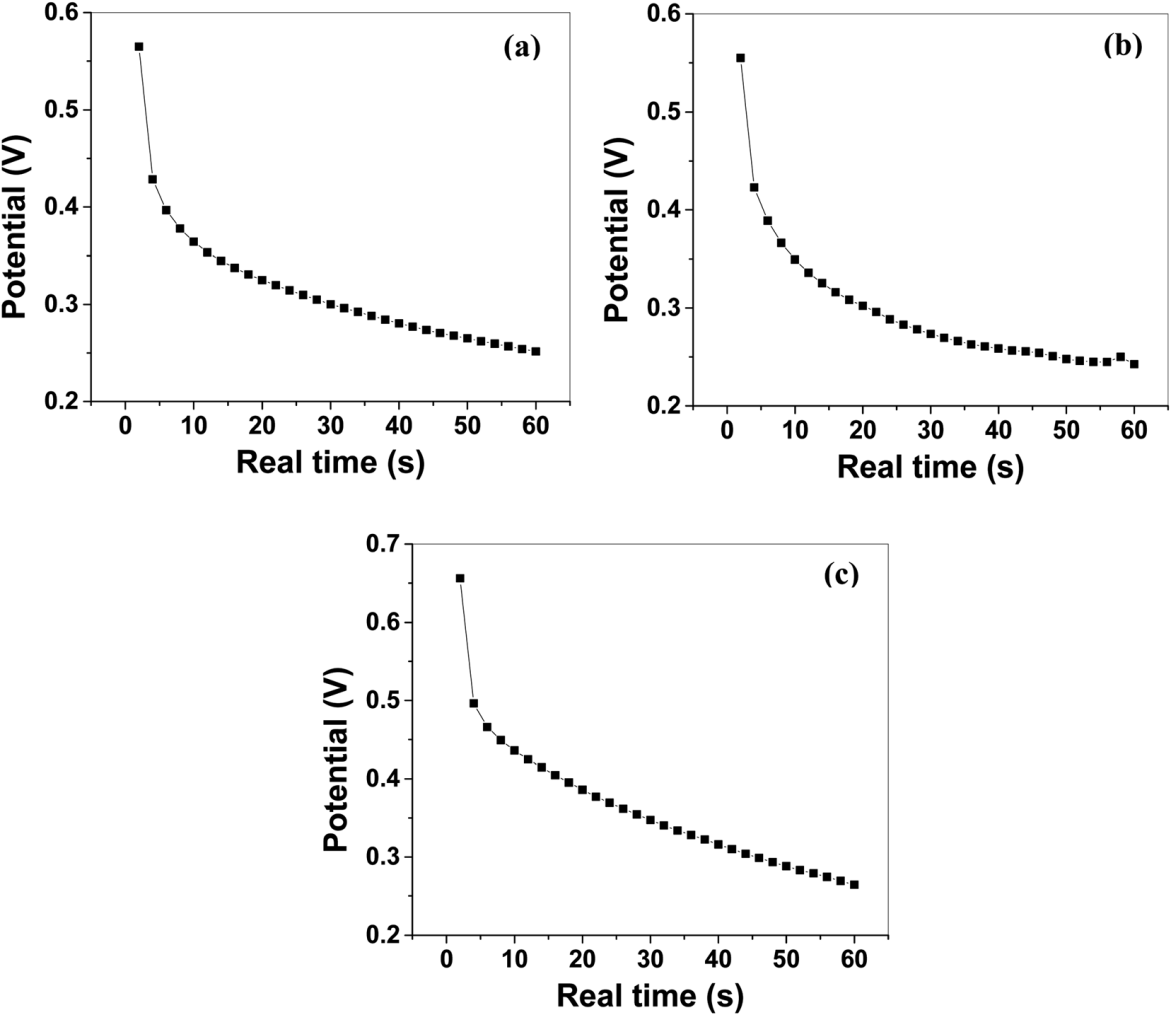
GCCD (galvanostatic cyclic charge–discharge): discharge curves of (a) MoS_2_ melt 5 mPP, (b) MoS_2_ melt 5 mP, and (c) MoS_2_ melt potential *vs.* real time (at charge density = 0.57 A g^−1^) in 1 M H_2_SO_4_ electrolyte.

The electrochemical stability and quantitative information regarding the electrochemical capacitance of MoS_2_ melt 5 mPP, MoS_2_ melt 5 mP, MoS_2_ melt, M5, mPP and mP electrodes were estimated by performing GCCD measurements in aqueous 1 M H_2_SO_4_ electrolyte as presented in Fig. 9a–c and S8a–c.† Consecutive charge–discharge cycles were performed repeatedly at 0.0025 A h capacity and 1 μA discharge current within the potential range of −0.4 to 1 V.

The *C*_sp_ calculated by using eqn (2) from GCCD measurements for MoS_2_ melt 5 mPP was 236.23 F g^−1^ at a current density of 0.57 A g^−1^. The *C*_sp_ calculated by GCCD measurement for MoS_2_ melt 5 mP was 117.15 F g^−1^ and for the MoS_2_ melt it was equal to 49.53 F g^−1^. The *C*_sp_ calculated by GCCD measurements for M5, mPP and mP electrodes were 90.54, 34.62 and 23.13 F g^−1^, respectively.

The energy density and power density of MoS_2_ melt 5 mPP were 64.31 W h kg^−1^ and 3858.42 W kg^−1^ at the current density of 0.57 A g^−1^, and were calculated by using the relations^92–94^ given in eqn (6) and (7), respectively.6
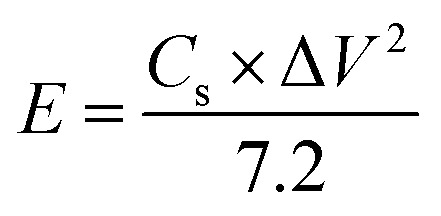
where *E* = specific energy density (W h kg^−1^), Δ*V* = potential window, *C*_s_ = specific capacitance.7
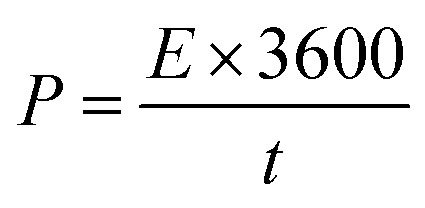
where *P* = power density (W kg^−1^), *E* = specific energy density (W h kg^−1^), *t* = discharge time.

The electrochemical results show that in MoS_2_ melt 5 mPP and MoS_2_ melt 5 mP composites, GNPs with polymers (mP and mPP) along with MoS_2_ melt enhance the electroactive sites for charge storage mechanisms as compared to the pristine MoS_2_ melt and provide shorter and continuous pathways for ion diffusion or charge flow. MoS_2_ melt 5 mPP shows better electrochemical performance (larger *C*_sp_ value) than MoS_2_ melt 5 mP, which shows that in hybrid composite form, mPP along with GNPs and MoS_2_ melt provides a more favourable configurational structure for the flow of charge inside the material as compared to the hybrid composite of mP along with GNPs and MoS_2_ melt.

## Conclusions

We have reported a cost-effective and facile method for attaining electrode material for supercapacitors with enhanced electrochemical storage performance. We synthesized MoS_2_ by the thermal decomposition of molybdenum dithiocarbamate in an inert environment and then used a liquid exfoliation method to form their composites with graphene nanoplatelets (GNPs) and polymers (polyaniline and polypyrrole).

The electrochemical results show that in the composites MoS_2_ melt 5 mPP and MoS_2_ melt 5 mP, GNPs with polymers (mP and mPP) along with MoS_2_ melt enhanced the electroactive sites for charge storage mechanisms as compared to the pristine MoS_2_ melt and provided shorter and continuous pathways for ion diffusion or charge flow. The MoS_2_ melt 5 mPP showed better electrochemical performance (larger *C*_sp_ of 236.23 F g^−1^ at a current density of 0.57 A g^−1^) than the MoS_2_ melt 5 mP (*C*_sp_ = 117.15 F g^−1^), which indicated that the hybrid composite from the mPP and GNPs and MoS_2_ melt provided a more favourable configurational structure for the flow of charge inside the material as compared to the hybrid composite of mP along with GNPs and MoS_2_ melt (*C*_sp_ = 49.53 F g^−1^). Electrochemical results revealed that the diffusion-controlled contribution to the total achieved a specific capacitance of MoS_2_/GNPs/conducting polymer-based composites is dominant, which is due to the combined effect of higher quantities of GNPs and MoS_2_ melt. At a current density of 0.57 A g^−1^, MoS_2_ melt 5 mPP provided a specific capacitance of 236.23 F g^−1^, energy density of 64.31 W h kg^−1^ and power density of 3858.42 W kg^−1^. The MoS_2_ melt 5 mPP composite electrode has a 91.87% capacitance retention after 10 000 charge–discharge cycles.

The enhanced electrochemical storage behaviour is credited to the synergistic effect between the constituent materials of hybrid composites. This research work puts forward a facile scalable development route for MoS_2_ and GNPs-based polymer hybrid composites as potential candidates for scale-up applications in the supercapacitor industry.

## Author contributions

MS and MAM: conceptualization, supervision, administration, resources, reviewing and editing, YK: supervision, administration and resources, SK: methodology and investigation, SN: methodology, investigation, formal analysis, writing – original draft preparation, validation and data curation.

## Conflicts of interest

There are no conflicts to declare.

## Supplementary Material

RA-013-D3RA04153K-s001
