# Supplementary material for: Molybdenum disulfide (MoS2) along with graphene nanoplatelets (GNPs) utilized to enhance the capacitance of conducting polymers (PANI and PPy)
Source: RSC Adv. 2023 Oct 2;13(41):28785–97. doi: 10.1039/d3ra04153k (PMC10543645; doi:10.1039/d3ra04153k)
Supplement: RA-013-D3RA04153K-s001 [file RA-013-D3RA04153K-s001.pdf]

Electronic Supplementary Material (ESI) for RSC Advances.
This journal is © The Royal Society of Chemistry 2023

Molybdenum Disulfide (MoS_2) along with Graphene Nanoplatelets (GNPs) Utilized to Enhance the Capacitance of Conducting Polymers (PANI and PPy)

Saima Nawaz[a,b], Yaqoob Khan[b], Sadia Khalid[b], Muhammad Azad Malik[c*], and M. Siddiq[a*]

a. Department of Chemistry, Quaid-i-Azam University, Islamabad 45320, Pakistan.
b. Nanoscience and Technology Department, National Centre for Physics, QAU Campus, Shahdra Valley Road, Islamabad 45320, Pakistan.
c. Department of Chemistry, University of Zululand, Private bag X1001 KwaDlangezwa, 3880, South Africa.

*Address correspondence to:
Email address: m_sidiq12@yahoo.com
Tel.: +92 5190642147
Email: malikmohammad187@gmail.com
Tel.: +44 7403781143

Material Characterization of $Mo(dtc)_4$

Synthesized *Tetrakis*(diethyldithiocarbamato) molybdenum (IV) *i.e.* $Mo(dtc)_4$ was characterized by elemental analysis, FT-IR and thermogravimetric analyses as discussed below.

Elemental Analysis

The theoretical values calculated (*i.e.* Anal. Calc.) for $Mo(dtc)_4$; $C_{20}H_{40}N_4S_8Mo$ are C, 34.9; H 5.9; N, 8.1% [1, 2] and in our synthesized $Mo(dtc)_4$ the values found are C, 34.9; H, 5.9; N, 8.1% using an organic elemental analyzer of Thermo Scientific Flash 2000.

Fourier Transform Infrared Spectroscopy (FT-IR)

FT-IR spectrum of $Mo(dtc)_4$ (Figure S1) is showing the peaks/bands at 2971, 2933, 2869, 1516, 1491, 1455, 1428, 1376, 1352, 1269, 1210, 1145, 1095, 1074, 1003 and 990 cm^{-1} which are consistent with earlier reports [1-3]. Three basic regions exist in infrared spectra of dithiocarbamato complexes;

- The first main region extents from 1450 to 1550 cm^{-1} and account for thioureide (NCSS) band which typically occurs betwixt C-N and C=N band. The absorption band at 1500 cm^{-1} is attributed as arising from polar structure —NCS_2.
- The second region (1070-930 cm^{-1}) is descriptive of mode of coordination in dithiocarbamato moiety (CSS).
- The third region around 350-400 cm^{-1} is attributed to M-S bonds. [1-3]

Please do not adjust margins

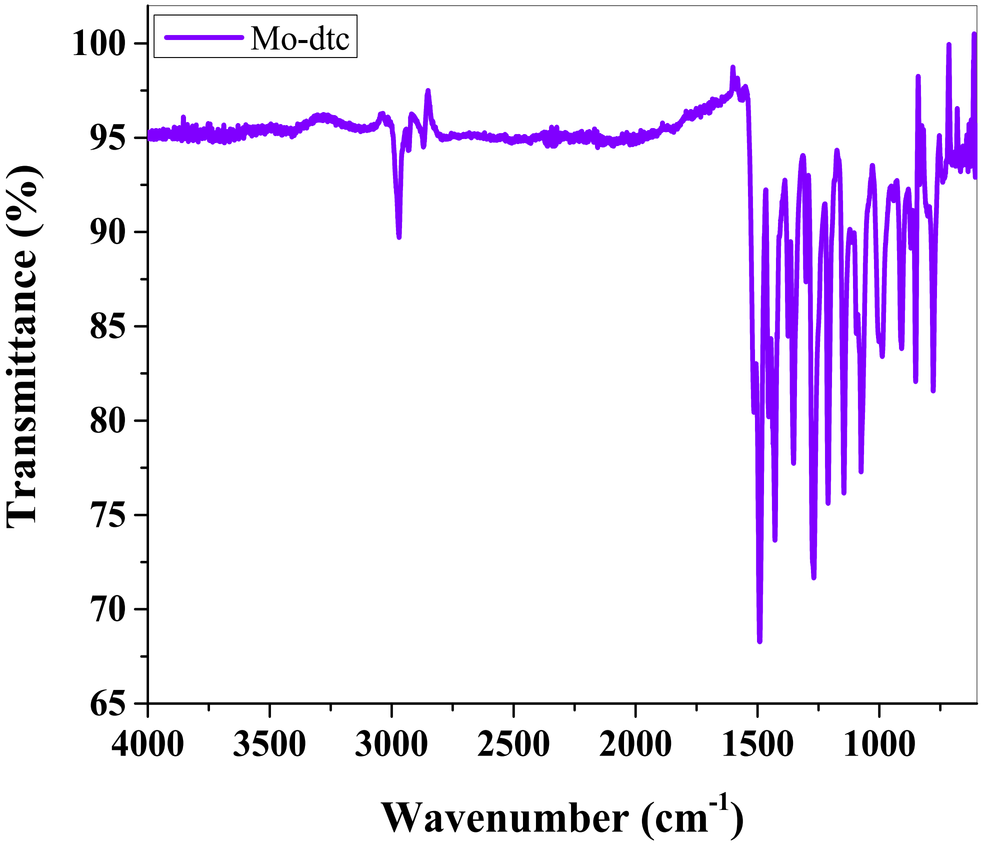

Figure S1: Fourier transform infrared (FT-IR) spectroscopy of Mo(dtc)$_4$.

Thermogravimetric Analysis (TGA)

Figure S2 displays the TGA of Mo(dtc)$_4$ under the environment of nitrogen. Three stages of decomposition take place at 151, 233, and 352 °C. The first -7.2 % decomposition step at 151 °C is due to the loss of ethylene (-4.0% calculated weight% loss) while -41.8% second step at 233 °C is due to two diethyldithiocarbamate molecules (-42.9% calculated weight% loss). The remaining material is a black solid with a residual weight percent of 28.3% after the third decomposition process (-22.7%). This thermal decomposition of Mo(dtc)$_4$ consequences to produce (23.5% calculated weight%) pure MoS_2, while aditional 4.8% is due to decomposed ligand contamination. [4]

Please do not adjust margins

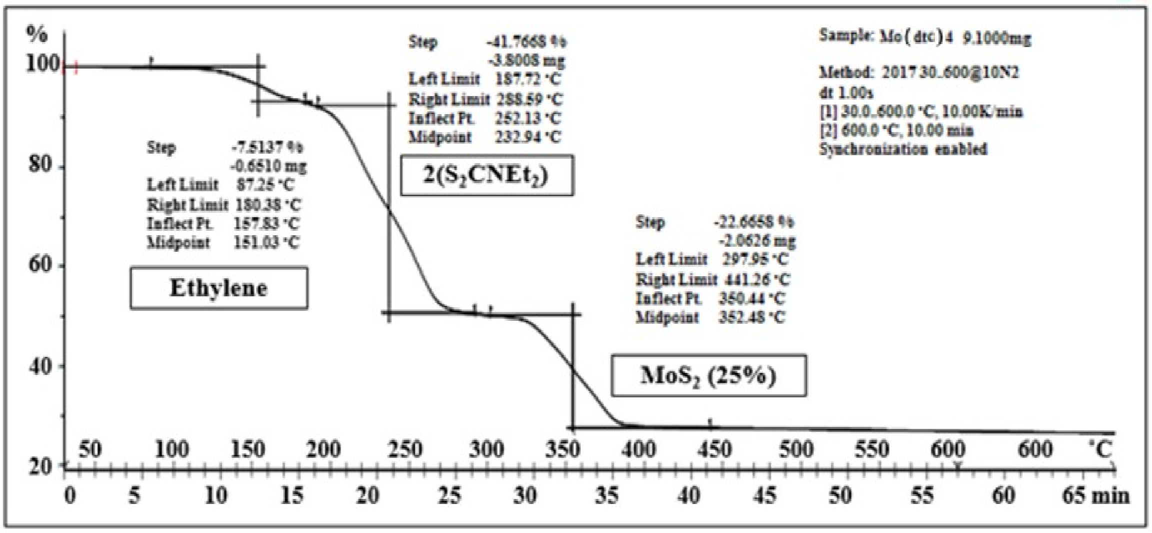

Figure S2: Thermogravimetric analysis (TGA) of Mo(dtc)$_4$.

Raman Analysis of MoS_2 melt

Raman spectra (Figure S3) indicates that thermolysis process under argon inert environment yields MoS_2 with A_{1g} out-of-plane mode centered at 361 cm^{-1} and E^1_{2g} in-plane mode seen at 334 cm^{-1} ($\Delta\nu$ = 27 cm^{-1}).[5] Phonon confinement may be the reason for softening of two modes and peaks broadening, which depicted that achieved resultant MoS_2 material is of few layers.[6, 7]

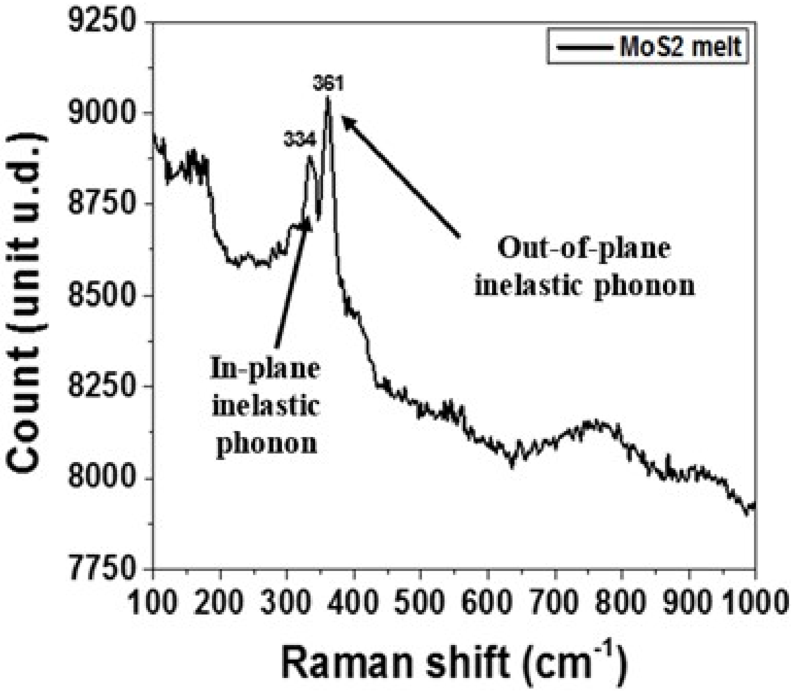

Figure S3: Raman analysis of MoS_2 melt.

Please do not adjust margins

Electrochemical Studies

Cyclic Voltammetry (CV)

CV at Various Scan Rates

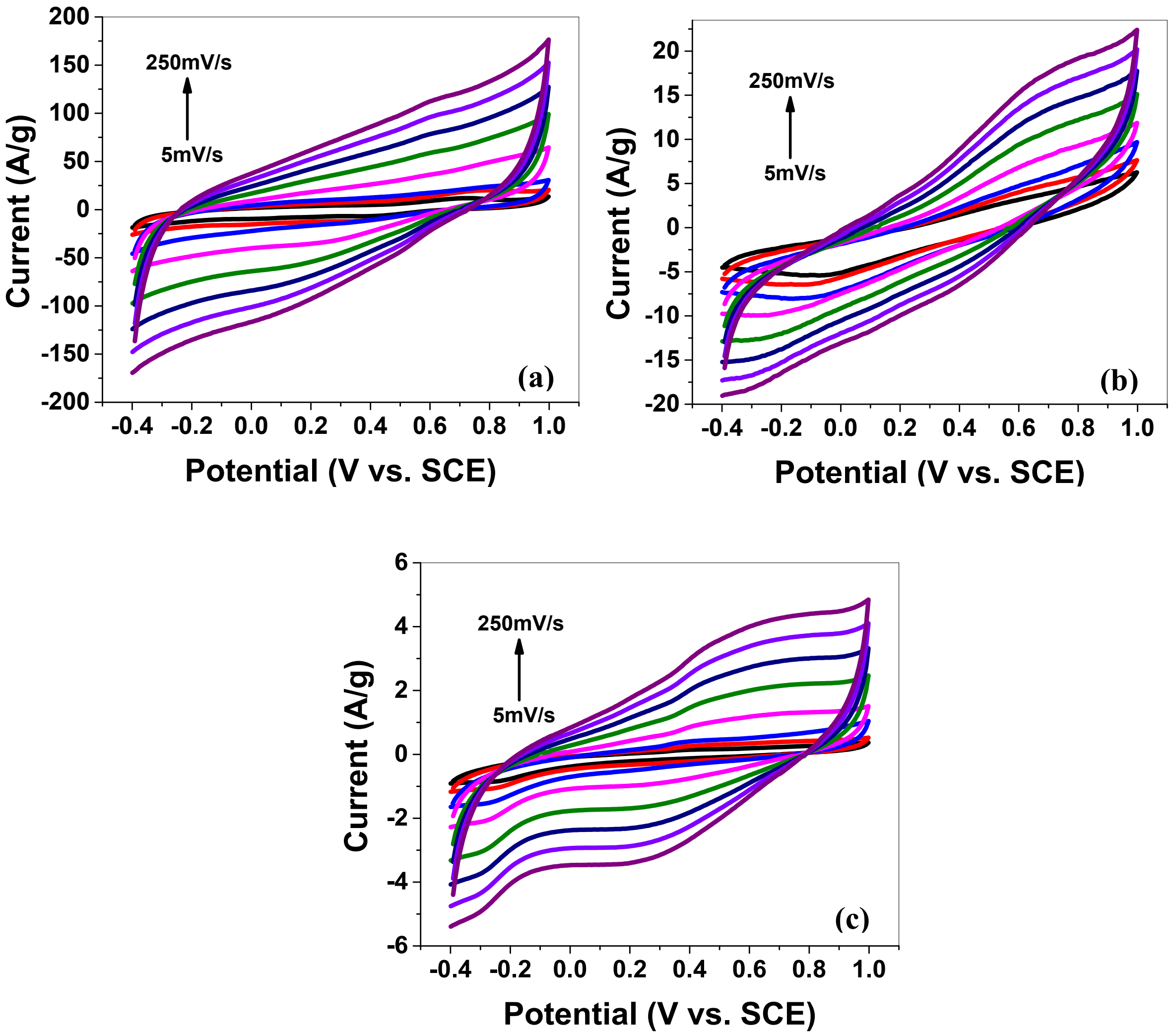

Figure S4: Cyclic voltammetry (CV) of (a) M5, (b) mPP and (c) mP at different scan rates on GCE in 1M H_2SO_4 vs. SCE.

Please do not adjust margins

Cyclic Stability for 50 Cycles of CV

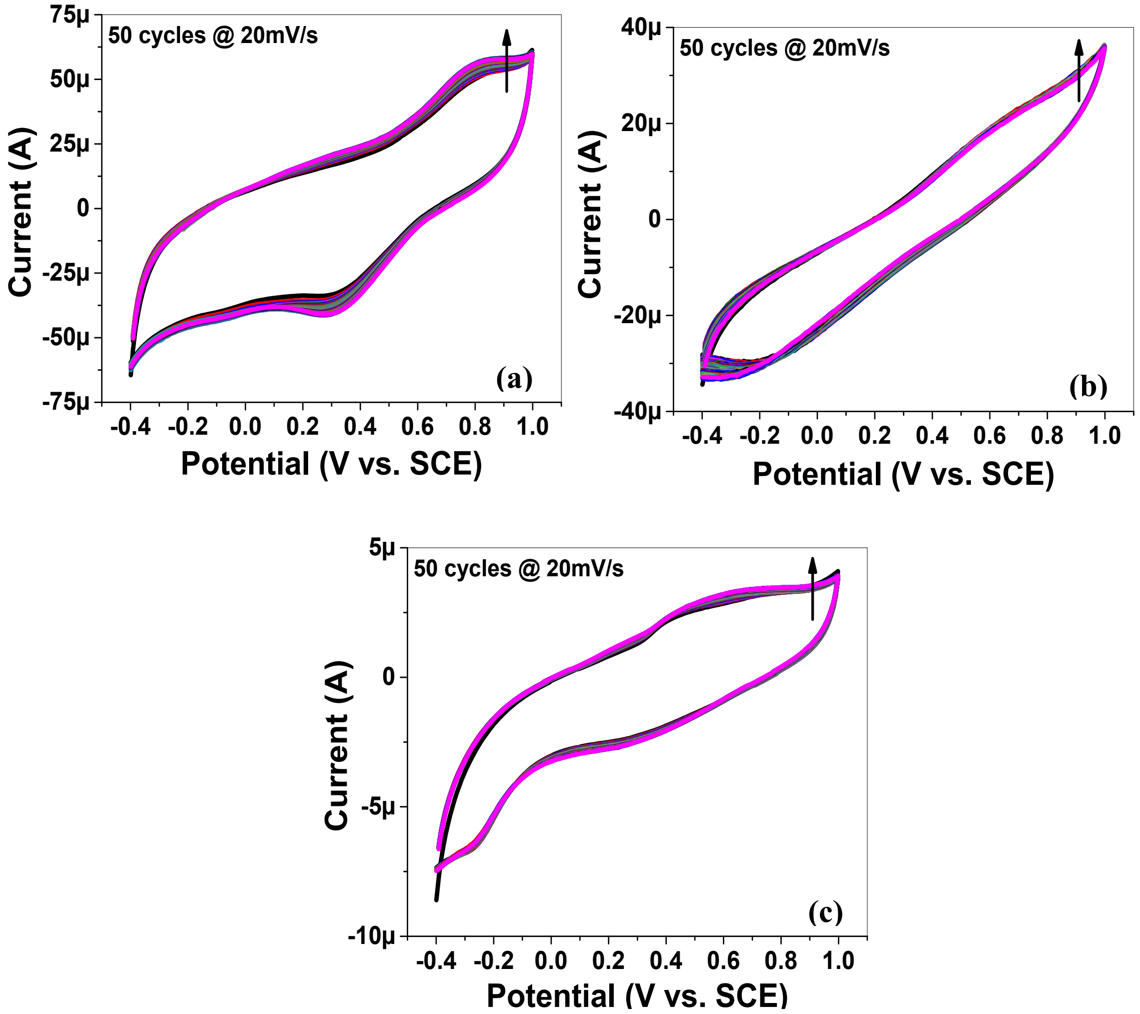

Figure S5: Cyclic voltammetry (CV): cyclic stability of (a) M5, (b) mPP and (c) mP at scan rate of 20mV/s for 50 cycles on GCE in 1M H_2SO_4 electrolyte vs. SCE.

Please do not adjust margins

EIS (Electrochemical Impedance Spectroscopy)

EIS Nyquist plots

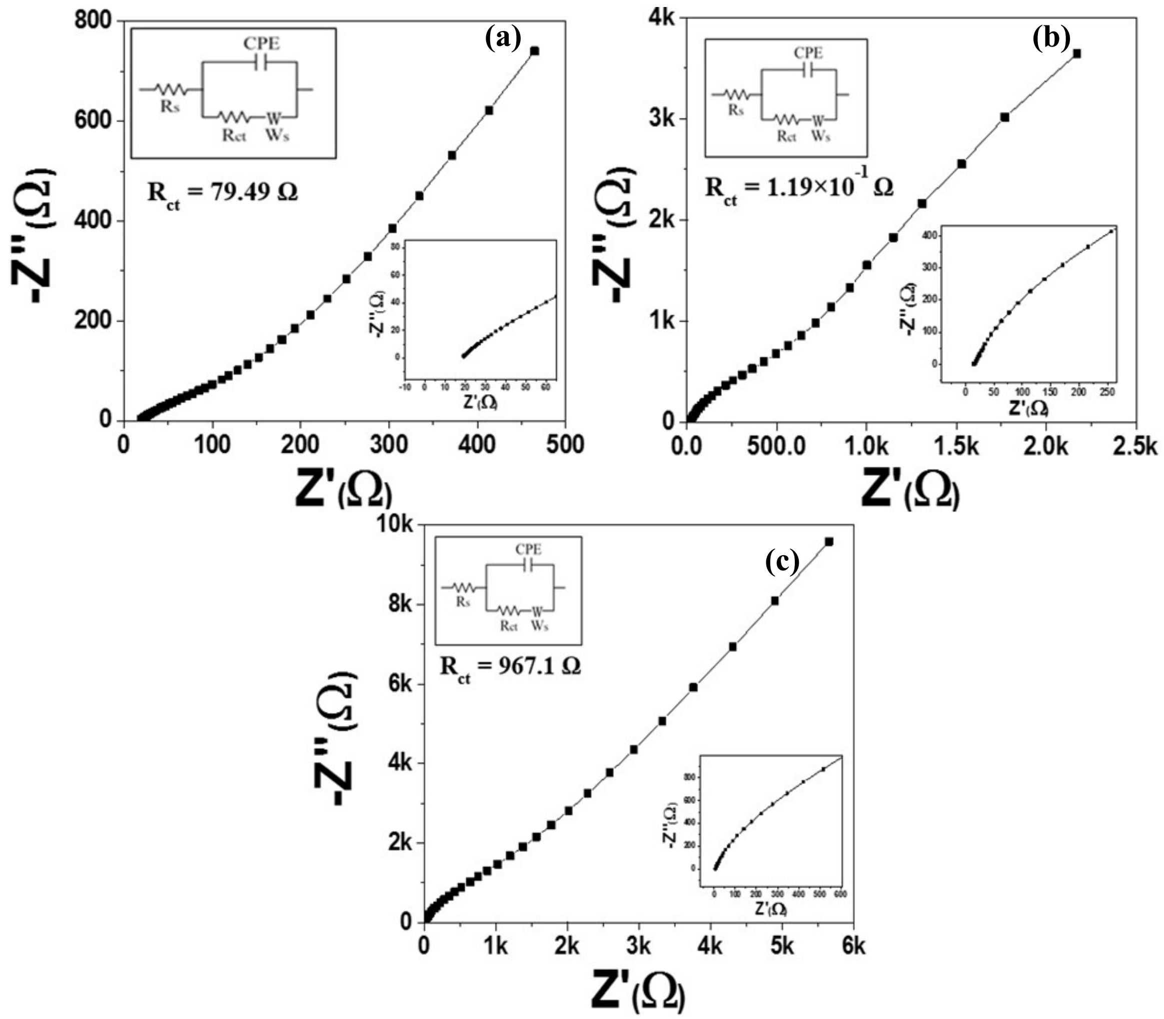

Figure S6: EIS Nyquist plots of (a) M5, (b) mPP and (c) mP vs. OC in 1M H_2SO_4 electrolyte with equivalent circuit diagram.

Please do not adjust margins

Please do not adjust margins

EIS Bode plots

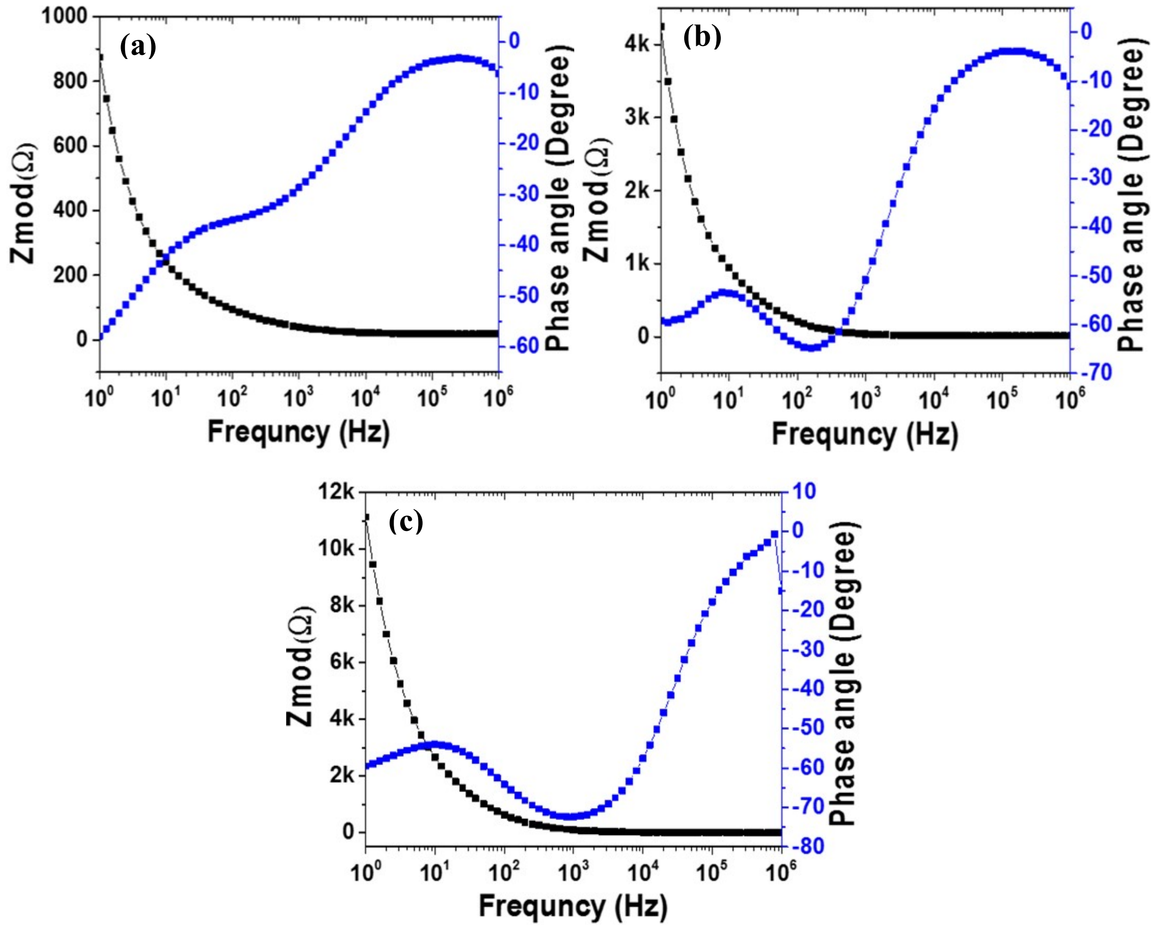

Figure S7: EIS (Electrochemical Impedance Spectroscopy): Bode plots of (a) M5, (b) mPP and (c) mP vs. OC (open circuit) at 10mv rms AC perturbation (in 1M H_2SO_4 electrolyte).

Please do not adjust margins

Please do not adjust margins

Galvanostatic Charge/Discharge (GCCD) Measurements

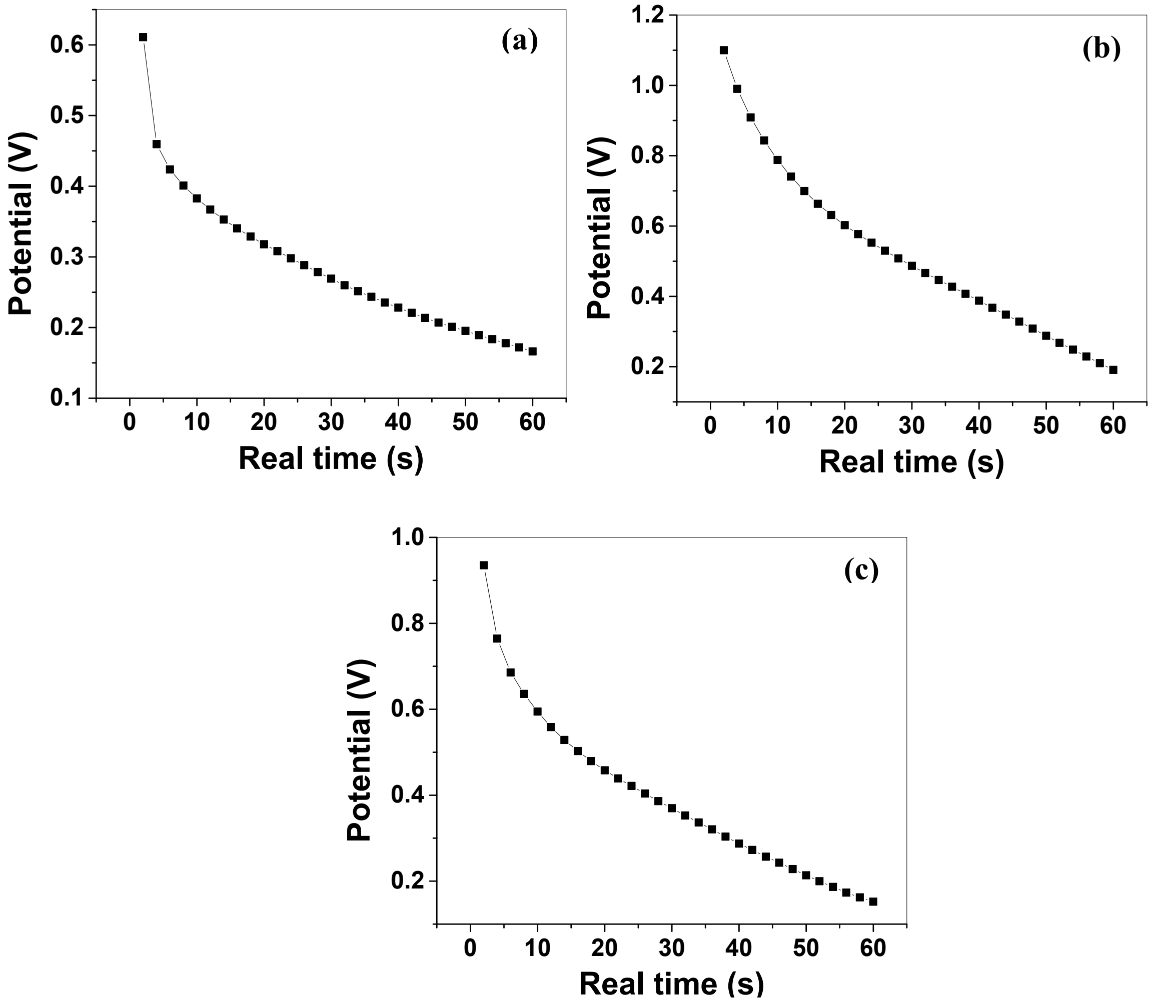

Figure S8: GCCD (Galvanostatic cyclic charge discharge): Discharge curve of (a) M5, (b) mPP and (c) mP Potential vs. real time (at charge density= 0.57 A/g) in 1M H_2SO_4 electrolyte.

This journal is © The Royal Society of Chemistry 20xx

Please do not adjust margins

Please do not adjust margins

Galvanostatic Charge/Discharge (GCCD) Measurements at different charge densities

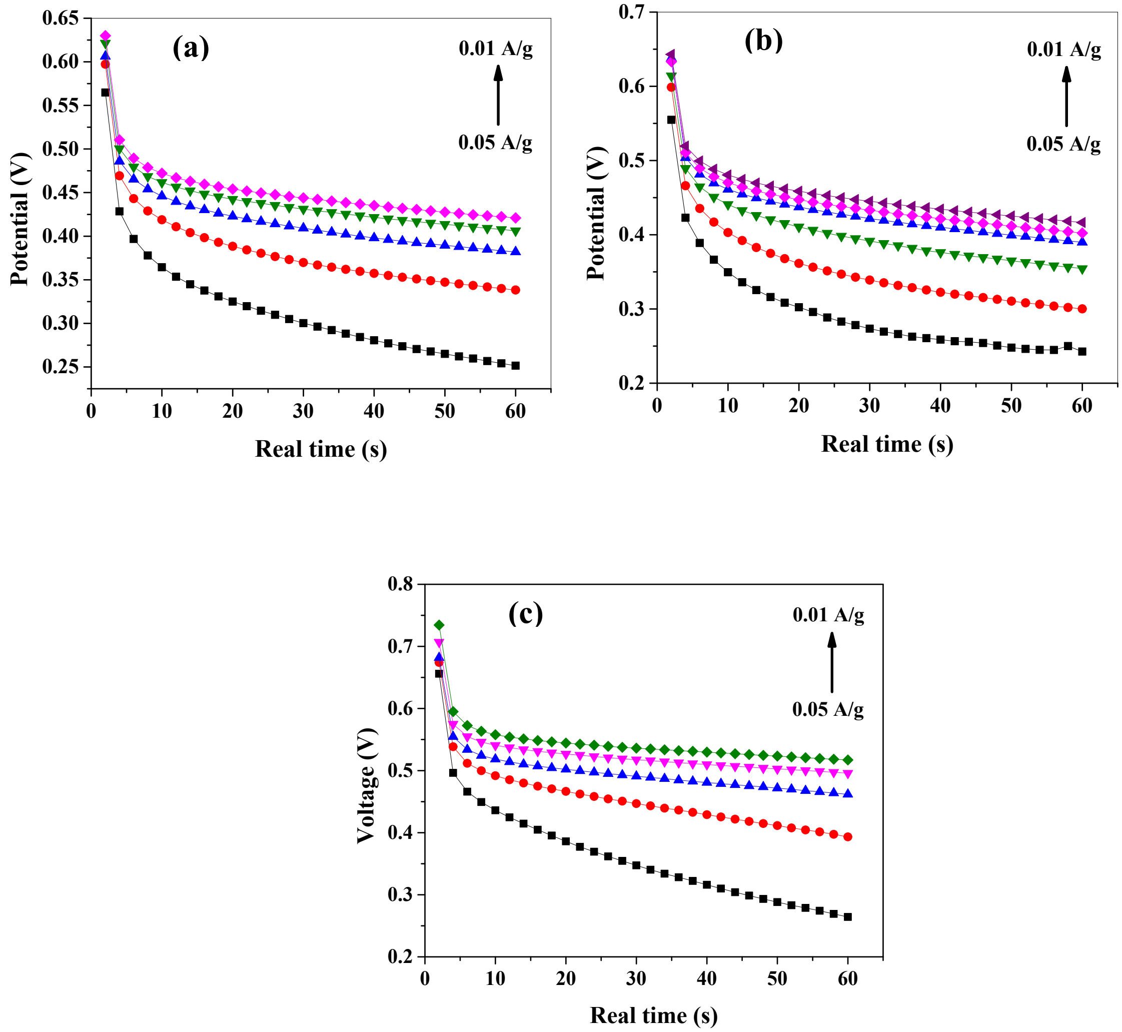

Figure S9: GCCD (Galvanostatic cyclic charge discharge): Discharge curve of (a) MoS_2 melt 5mPP, (b) MoS_2 melt 5mP and (c) MoS_2 melt Potential vs. real time at different charge densities in 1M H_2SO_4 electrolyte.

This journal is © The Royal Society of Chemistry 20xx

Please do not adjust margins

Please do not adjust margins

Galvanostatic Charge/Discharge (GCCD) Capacitance retention of MoS_2 melt 5mPP vs. cycle number for 10,000 cycles

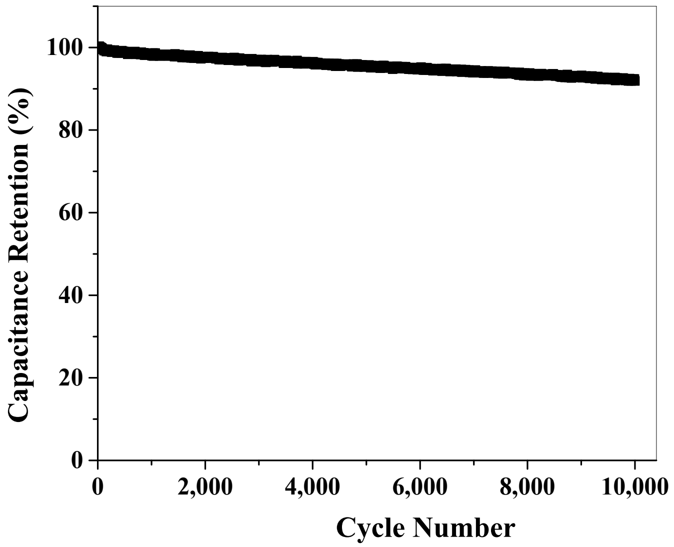

Figure S10: GCCD: Capacitance retention of MoS_2 melt 5mPP vs. cycle number for 10,000 cycles (in 1M H_2SO_4 electrolyte).

Capacitance retention of MoS_2 melt 5mPP for 10,000 charge-discharge cycles in 1M H_2SO_4 electrolyte is shown in Figure S10. The composite demonstrates improved charge storage performance and excellent cyclic stability (91.87% capacitance retention at 0.57 A/g after 10,000 cycles).

This journal is © The Royal Society of Chemistry 20xx

Please do not adjust margins

Please do not adjust margins

Raman Analysis of M5-Graphene nanoplatelets

Raman spectra for M5-Graphene nanoplatelets is shown in Figure S11.

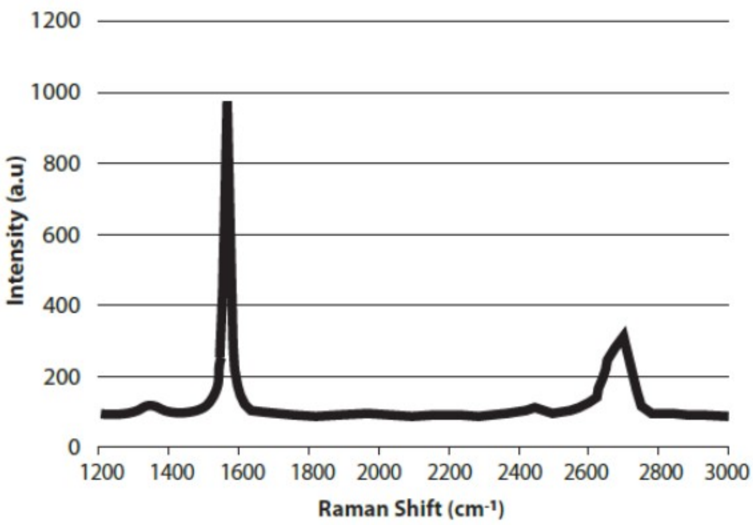

Figure S11: Raman analysis of M5.

Please do not adjust margins

Please do not adjust margins

Notes and References

1. D. J. Lewis, A. A. Tedstone, X. L. Zhong, E. A. Lewis, A. Rooney, N. Savjani, J. R. Brent, S. J. Haigh, M. G. Burke, C. A. Muryn, J. M. Raftery, C. Warrens, K. West, S. Gaemers, and P. O'Brien, Chemistry of Materials, 2015, 27, 1367-1374.
2. E. P. C. Higgins, S. G. McAdams, D. G. Hopkinson, C. Byrne, A. S. Walton, D. J. Lewis, and R. A. W. Dryfe, Chemistry of Materials, 2019, 31, 5384-5391.
3. A. Nieuwpoort, Dithiolato complexes of molybdenum and tungsten, 1975, Katholieke Universiteit Nijmegen (Netherlands).
4. M. N. McCain, B. He, J. Sanati, Q. J. Wang, and T. J. Marks, Chemistry of Materials, 2008, 20, 5438-5443.
5. C. N. R. Rao and A. Nag, European Journal of Inorganic Chemistry, 2010, 2010, 4244-4250.
6. C. Lee, H. Yan, L. E. Brus, T. F. Heinz, J. Hone, and S. Ryu, ACS Nano, 2010, 4, 2695-2700.
7. N. Zeng, D. G. Hopkinson, B. F. Spencer, S. G. McAdams, A. A. Tedstone, S. J. Haigh, and D. J. Lewis, Chemical Communications, 2019, 55, 99-102.

This journal is © The Royal Society of Chemistry 20xx

Please do not adjust margins
